# Probing the interactions between hardness and sensory of pistachio nuts during storage using principal component analysis

**DOI:** 10.1002/fsn3.1124

**Published:** 2019-07-22

**Authors:** Mohammad B. Habibi Najafi, Anders Leufven, Mohammad Reza Edalatian Dovom, Naser Sedaghat, Amir Pourfarzad

**Affiliations:** ^1^ Department of Food Science &Technology Ferdowsi University of Mashhad (FUM) Mashhad Iran; ^2^ Norwegian Institute of Food Fisheries and Aquaculture Research Tromso Norway; ^3^ Department of Food Science and Technology, Faculty of Agricultural Sciences University of Guilan Rasht Iran

**Keywords:** packaging, pistachio nuts, principal component analysis, sensory and hardness, storage time

## Abstract

Instrumental hardness and sensory characteristics of pistachio nuts such as flavor, texture, and overall acceptability were measured in three different packages (atmosphere, vacuum, and oxygen scavenger), at three different conditions temperatures (20, 35, and 50˚C) during 12 weeks’ storage. Results showed that the effect of temperature, storage time, and interaction effects of packaging and temperature, and packaging and storage time, and packaging and temperature and stoarge time on instrumental hardness were significant (*p* < 0.01). In sensory evaluation, the effect of treatments on sensory attributes was not significant. Instrumental hardness and sensory texture had a high correlation with each other. Principal component analysis (PCA) of the data obtained for the samples permitted the reduction of the variables to two principal components, which together explained 60.7% of the total variability.

## 1 INTRODUCTION

Pistachio nuts (*Pistacia Vera L*.) are grown mainly in Iran, USA, Syria, Turkey, Greece, and Italy. It is one of the most popular nuts in the world with its high nutritional value and unique flavor as a snack and a food ingredient. It contains around 23% proteins, 19% carbohydrate, and 5% moisture. Pistachio is an edible nut from pistachio tree. That is mainly cultivated in warm and dry areas of the Middle East, Mediterranean countries, and the United States. The main region of pistachio cultivation in Iran includes Rafsanjan, Sirjan, Damghan, Ghazvin, Tabriz, Torbat‐e‐ Heydarieh, Tabas, Birjand, and Shiraz (Gamlı & Hayoğlu, 2007; Garcia, Agar, & Streif, 1992). The kernels are a rich source of oil (50%–60%) and contain linolenic and linoleic fatty acids. Dried raw pistachio with 4%–6% moisture content is very stable and can be stored more than 12 months in 20°C without any quality alteration (Kader, Heintz, Labavitch, & Rae, 1982; Maskan & Karataş, 1998).

Quality of many foods and drinks decreases during storage, and the rate of this reduction depends mainly on the type of food. Lipid oxidation is a major cause of quality deterioration in dehydrated foods (Barden, 2014). The degree of unsaturation or polyunsaturation, tocopherol, carotene, chlorophyll, mineral, moisture content, and temperature affect primarily lipid oxidation and storage stability of intermediate moisture foods during storage (Damodaran, Parkin, & Fennema, 2007). In the presence of oxygen, oxidative reactions are usually of the greatest importance and storage life is then limited by the development of oxidative rancidity in fat (Maskan & Karataş, 1998).

Oxidation reaction is commonly controlled by reducing the oxygen concentration in the atmosphere of storage of dry or intermediate moisture foods using vacuum or nitrogen filling (Kacyn, Saguy, & Karel, 1983). Also, CO_2_ filling is applied for fresh fruits and vegetables, meat, chicken, fish, and bakery products in order to avoid microbial growth and lipid oxidation (Hotchkiss, 1988; Lioutas, 1988). The gas concentration of O_2_, CO_2_, or N_2_ should be controlled for each individual product. Researchers reported that, for a model consisting of Avicel microcrystalline cellulose and methyl linoleate, an O_2_ concentration of below 2% affects the rate constant dramatically with the rate decreasing sharply as the O_2_ is decreased (Kacyn et al., 1983). Some researchers used 0.5% O_2_ in their study for an intermediate moisture food (Waletzko & Labuza, 1976). However, Brecht (1980) proposed 0% O_2_ and 100% CO_2_ for tree nuts.

Microbial growth and other deteriorative chemical reactions may be facilitated by high concentrations of oxygen which is present in food packages and finally results in the shelf‐life reduction of foods (Ozdemir & Floros, 2004). Thus, limit and control of oxygen concentration in food packages are essential to restrict the rate of such detrimental and spoilage reactions in foods. Systems which are absorbing oxygen provide an alternative to vacuum and gas flushing technologies for extending product quality and shelf life (Ozdemir & Floros, 2004). Concepts such as iron powder oxidation, ascorbic acid oxidation, photosensitive dye oxidation, enzymatic oxidation (e.g., glucose oxidize and alcohol oxidize), and unsaturated fatty acid oxidation (e.g., oleic or linolenic acid) are utilized in oxygen scavenging technologies (Floros, Dock, & Han, 1997). The oxygen scavenging component of a package can be presented in the form of a sachet, label, film (incorporation of scavenging agent into the packaging film), card, closure liner, or concentrate (Kerry, Ogrady, & Hogan, 2006; Suppakul, Miltz, Sonneveld, & Bigger, 2003).

The objective of this investigation was to determine the effect of temperature, storage period, and packaging conditions on instrumental hardness and sensory evaluation (flavor, texture, and overall acceptability) and evaluate the correlation between instrumental hardness and sensory scores of Ohadi pistachio nut variety using PCA.

## 2 MATERIALS AND METHODS

### 2.1 Materials

#### 2.1.1 Pistachio nuts

The Ohadi variety of Iranian pistachio nuts (*Pistacia vera L.)* was obtained from Faizabad Pistachio Factory, Khorasan, Iran. The average moisture content of the nuts was determined to be 3.8%, using the oven‐drying method. Samples were placed in sealed plastic bags and kept at 0–1ºC until the day of experiments.

#### 2.1.2 Packaging material

Transparent plastic multilayer pouches (PE/PA/PE) with a thickness of 80 µm, manufactured by Henkelman (Hertogenbosch, the Netherlands), were used for storage of pistachio nuts at different conditions.

### 2.2 Methods

#### 2.2.1 Sample preparation

100 g raw dried pistachio nuts were dispensed into transparent plastic multilayer pouches (22 × 17.5 cm). The plastic pouches were then sealed by a sealing chamber vacuum machine (Model 200A, Henkelman, Holland) under three packaging conditions, atmosphere, vacuum, and oxygen scavenger (AGELESS^®)^. This oxygen scavenger has designed to be used with dehydrated or dried foods. All samples were prepared in triplicates for accelerated storage conditions.

#### 2.2.2 Accelerated storage conditions

Plastic pouch samples were held in controlled temperatures at 20, 35, and 50°C for 4, 8, and 12 weeks. The beginning of the storage period was assigned as time zero. Storage temperatures and time were selected based on dehydrated food shelf‐life studies (Waletzko & Labuza, 1976).

#### 2.2.3 Hardness measurement (texture analyzer)

The mechanical properties (hardness) of raw dried pistachio nuts were determined by a compression test using a texture analyzer QTS‐25 (CNS Farnell, Boreham Wood, Hertfordshire, England, WD61WG). Six pistachio nuts of each sample were tested individually by use of Ottawa cell (4940‐33, plunger 25 with Blank plate). The test method was defined to measure the force required for penetration of the probe at a velocity of 50 mm/min into the pistachio samples. The probe descended 5 mm, and the trigger force was set at 10 g.

Only pistachio nuts with no sign of skin breakage and defect were selected for testing. The average value was calculated. The collected data were then analyzed to study the correlation for sensory scores using standard regression analysis.

#### 2.2.4 Sensory evaluation

Sensory evaluation was performed using descriptive analysis method (Shakerardekani, 2017). Ten panelists that consist of staff members and graduate students with ages between 20 and 30 years were participated. A 5‐point hedonic scale was used (5 = excellent, 4 = good, 3 = fair, 2 = poor, and 1 = bad). Samples were presented at room temperature in individual booths, each panelist received six nuts per sample in colorless transparent plastic dish coded with 3‐digit random numbers, fresh water was provided to drink between evaluations. Each panelist was asked to score three main components of raw dried pistachio nuts, namely taste, texture, and overall acceptability in terms of the degree of liking each sample. The evaluation was performed with all samples at 0, 4, 8, and 12 weeks’ intervals.

### 2.3 Statistical analysis

Data analysis and evaluating the effect of temperature, storage period, and packaging conditions on instrumental hardness and sensory parameters of samples were carried out using a factorial completely randomized design. After analysis of variance, Duncan's multiple range test was used to investigate the significant differences between the means of the data with 95% confidence. PCA was performed on texture and sensory data sets. Statistical analysis of the data was done using Minitab software (Minitab 15, Minitab Inc., State College, PA, USA).

## 3 RESULT AND DISCUSSION

### 3.1 Measurement of instrumental hardness of pistachio texture

**Figure 1:**
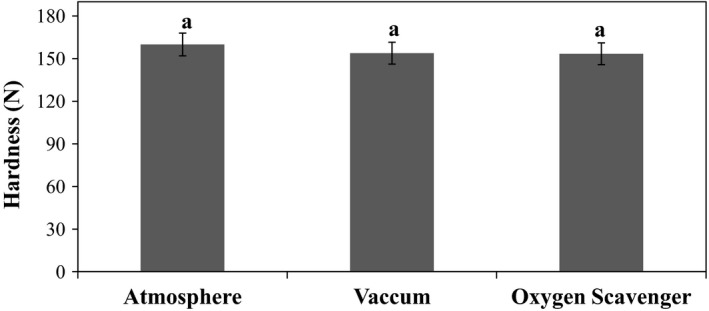
Effect of packaging system on the instrumental hardness of raw dried pistachio nuts

Results of analysis of variance (ANOVA) (Table 1) indicated that effects of temperature and storage time and also interaction of some factors (packaging systems × temperature, packaging systems × storage, and packaging systems × temperature ×storage time) on the instrumental hardness of pistachio were significant (*p* < 0.01). The effect of packaging systems was not significant (*p* > 0.05). However, LSD test revealed that increasing trend of pistachio hardness as follows (Figure 1):

Normal air packaging > vacuum packaging > oxygen scavenger packaging.

**Table 1:** Analysis of variance for the effect of packaging system, temperature, and time on the pistachio characteristics

Source	*df*	Hardness—instrumental		Hardness—sensory		Flavor		Total acceptance	
		Sum of squares	*F* value	Sum of squares	*F* value	Sum of squares	*F* value	Sum of squares	*F* value
Packaging (A)	2	4,836,600	0.27^ns^	0.45	0.23^ns^	3.05	1.97^ns^	1.67	1.12^ns^
Temperature (B)	2	376,621,904	20.73***	3.23	1.64^ns^	1.83	1.18^ns^	1.09	0.73^ns^
Time (C)	2	121,588,190	6.69**	2.25	1.14^ns^	0.56	0.36^ns^	1.62	1.09^ns^
A × B	4	349,679,705	9.62***	2.01	0.51 ^ns^	2.21	0.72 ^ns^	2.11	0.71 ^ns^
A × C	4	222,574,873	6.12**	1.66	0.42^ns^	1.41	0.46^ns^	0.04	0.01^ns^
B × C	4	67,413,043	1.85^ns^	1.48	0.38^ns^	2.50	0.81^ns^	1.16	0.39^ns^
A × B×C	8	605,228,601	8.33***	2.67	0.34^ns^	4.85	0.78^ns^	2.71	0.46^ns^
Error	27	245,323,312		239.50		187.90		180.40	
Total	53	1,993,266,229		253.26		204.33		190.80	

Note

ns: no significant effect at level < 0.05.

* *p* < 0.05.

** *p* < 0.01.

*** *p* < 0.001.

**Figure 2:**
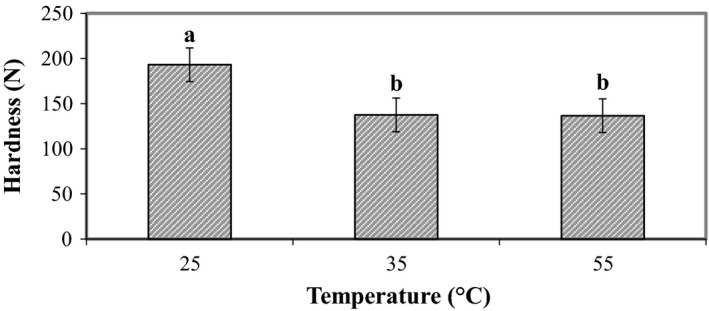
Effect of temperature on the instrumental hardness of raw dried pistachio nuts

This implies that oxygen and normal air make the pistachio harder so that, with reduction or elimination of oxygen in packaging, the pistachio hardness has been declined. Effect of temperature was significant (*p* < 0.01) on the instrumental hardness of pistachio (Figure 2), so that hardness declined with increasing temperature.

Influence of storage time was significant on pistachio hardness, and with time, hardness experienced a declining trend.

**Figure 3:**
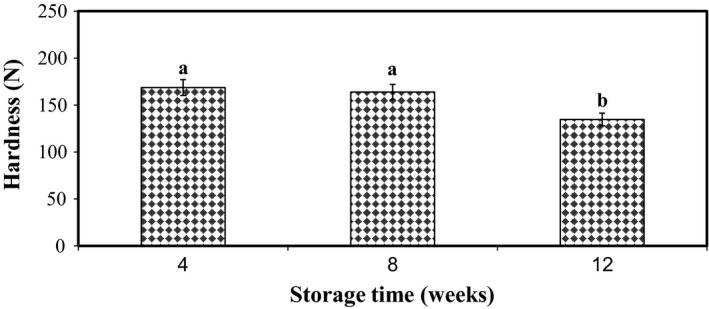
Effect of storage time on the instrumental hardness of raw dried pistachio nuts

As depicted in Figure 1, with increasing oxygen concentration, the hardness increased, and with decreasing or eliminating the oxygen level in packaging with vacuum or oxygen scavenger, the pistachio hardness decreased. Increase in temperature made hardness decrease (Figure 2). The hardness level showed significant (*p* < 0.01) decrease during storage time (Figure 3), which might be due to the effect of high temperature and long storage time on the excess of decay reaction that results in softening of texture. Koyuncu (2004) showed that during storage of hazelnuts, the total fat content increased, and the palmitic and oleic acid content of the oil increased. The effect of storage of shelled and unshelled hazelnuts on the total fat content was significant.

**Figure 4:**
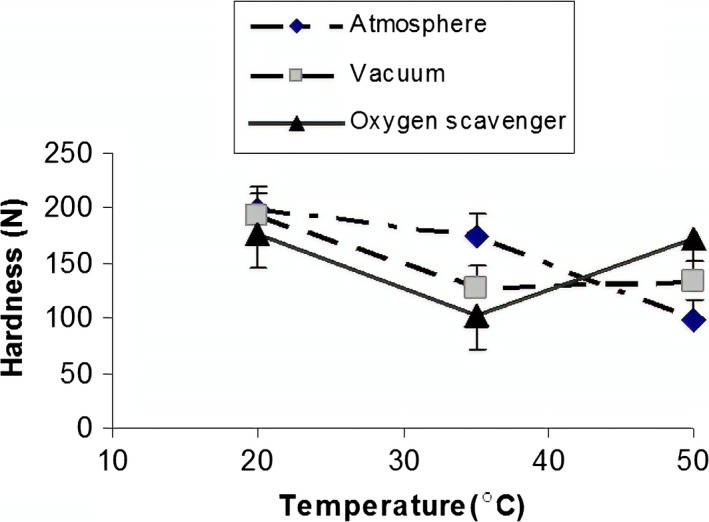
Effect of packaging and temperature on instrumental hardness of pistachio

**Figure 5:**
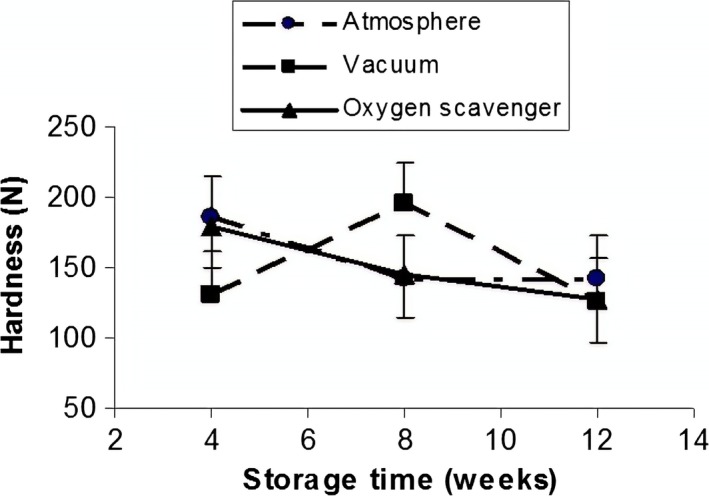
Interaction effect of packaging system and storage time on hardness of raw dried pistachio nuts

Results of interaction effect of packaging system/temperature and packaging system/storage time on the hardness of raw dried pistachio nuts are shown in Figures 4 and 5.

It is seen from Figure 4 that by changing the packaging system from atmospheric to vacuum and to oxygen scavenger, the hardness of samples decreased with temperature increase from 20 to 35^○^C. However, by increase the temperature up to 50^○^C, hardness at vacuum and oxygen scavenger packaging systems was increased, whereas hardness at the atmospheric packaging system was decreased. This difference in hardness might be due to the effect of oxygen which can cause increase in lipid oxidation and subsequently change in texture. Decreasing the hardness due to use of higher temperature has been shown by some researchers for sesame seeds (Kahyaoglu & Kaya, 2006). As depicted in Figure 4, with increasing storage temperature, the hardness of texture reduced in all three packaging types, which can be due to softening of tissue (texture) as a result of activity of some decomposing enzymes. In oxygen scavenging packaging system, increase in hardness can be seen with temperature from 35 to 55°C due to lignin formation at this state (González‐Aguilar, Ayala‐Zavala, Ruiz‐Cruz, Acedo‐Félix, & Dıaz‐Cinco, 2004).

The reason for desirable stability of pistachio and its shelf life at different storage conditions may be attributed to high oleic acid content in its lipid compositions. Garcia et al. (1992) demonstrated that oils with high oleic acid content (such as pistachio oil) have an acceptable shelf life, these types of oils are considered useful from nutritional point of view, and their consumption leads to cholesterol reduction.

Interaction effect of packaging systems and storage time was significant on instrumental hardness of pistachio (Figure 5).

Figure 5 shows that pistachio hardness decreases with increasing storage time in atmospheric and oxygen scavenger packaging systems. However, with increasing storage time in vacuum packaging, pistachio hardness increased up to 8 weeks and after that decreased. This trend could be related to the presence of gas on hardness in atmospheric and oxygen scavenger packaging, while there was no gas in vacuum packaging. O'Connor‐Shaw, Roberts, Ford, and Nottingham (1994) found that fresh‐cut papaya texture declined significantly after 2 days of storage at 13^○^C. Barry‐Ryan and O’Beirne (2000) reported that firmness values for shredded carrots increased slightly in MAP during storage after 4 days at 3^○^C (Barry‐Ryan & O’Beirne, 2000). Jangchud, Puchakawimol, and Jangchud (2007) showed that the hardness of coconut meat from unwrapped, film‐wrapped, and vacuum‐packed coconut was significantly decreased (*p* < 0.05) on days 7 (8.56 N), 11 (7.56N), and 28 (7.54 N) of storage, respectively (Jangchud et al., 2007).

Interaction effect of packaging systems, temperature, and storage time on instrumental hardness of pistachio was significant (*p*‐value < 0.01).

In sensory evaluation of raw dried pistachio nuts (flavor, texture, and overall acceptability), analysis of variance (ANOVA) showed that the effect of all factors (packaging, temperature, and storage time) and their interaction were not significant (*p* > 0.05) (data not shown). Maskan and Karataş (1999) showed that the flavor and color of pistachio samples stored in the CO_2_ atmosphere were better than those of the control stored in atmospheric systems (Maskan & Karataş, 1999).

It should be pointed out that strong correlation was obtained from results of sensory and instrumental evaluation that means the effect of packaging system on sensory hardness of pistachio was the same order as of instrumental hardness which was as follow:

Atmosphere (normal air) > vacuum > oxygen scavenger.

With temperature increase, pistachio hardness also decreased. This finding was in agreement with Sedaghat, Mortazavi, Nasiri‐Mahalati, and Davari Nejad (2006).

Sensory hardness of pistachio texture decreased with progress of storage period. Results obtained for flavor property were in agreement with results of Sedaghat et al. (2006).

Flavor acceptability decreased with increase in the temperature during storage period, which might be related to the effects of storage time on fatty acid deterioration/oxidation producing off‐flavor and rancidity of pistachio oil.

Blakistone and Blakistone (1998) reported that use of ozone gas is the most common method for reduction of residual oxygen in packs containing pistachio nuts and dried fruits, and this method can be also successfully applied for raw and roasted pistachio.

Application of less oxygen concentration and cold storage conditions can prolong the shelf life of this product. High oleic acid content, natural antioxidant such as tocopherols, and low moisture are considered as effective factors in extending shelf life and stability of raw dried pistachio (Garcia et al., 1992). Regarding the stability and shelf life of pistachio nut, other researchers have presented similar reports (Kader et al., 1982). Maskan and Karataş (1998) reported that difference in peroxide value of pistachio nut was not significant during different storage conditions for six months and also reported that pistachio nuts possess acceptable stability.

Maskan and Karataş (1999) showed that lower rate of peroxide formation was seen in samples stored under CO_2_ than those stored under oxygen at similar temperature. On the other hand, they showed that flavor and color of samples stored under CO_2_ were better than control samples and samples stored in air system (Maskan and Karatas, 1999).

Probably, as temperature increases, the solubility of CO_2_ in oily parts and moisture of pistachio nut becomes ineffective. Gas solubility in water is inversely proportional to storage temperature, and therefore, low temperatures have a synergistic effect on its action. In low temperatures, CO_2_ has a protective effect on lipid oxidation. With increase in temperature up to 30°C, the effect of CO_2_ declines and ratio of *K* values of CO_2_ and air system reaches to 1; this means that there is no significant difference between storage in air and CO_2_. Nonprotective effect of CO_2_ in high temperature can be explained by this fact that solubility of CO_2_ in oil declines at high temperature. In contrast to CO_2_, the solubility of H_2_, O_2_, and N_2_ in oil increases with temperature increase. The presence of oxygen in storage atmosphere, even in trace amounts at high temperature, causes an increase in the reaction rate in spite of the presence of CO_2_ in high amounts (Maskan & Karataş, 1998).

**Figure 6:**
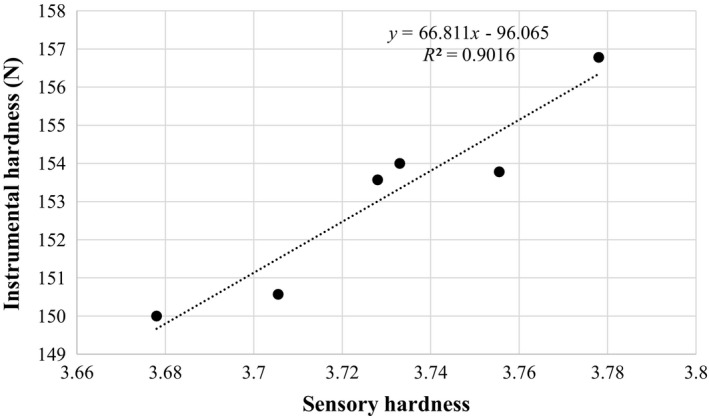
Relationship between instrumental and sensory hardness

Correlation between sensory evaluation (texture) and instrumental texture profile (hardness) of raw dried pistachio nuts is shown in Figure 6. Positive and powerful correlation of 0.9016 was observed between sensory textures with the texture analyzer measurement (hardness) of the compression force. Ho and Chokyun (1978) showed a good correlation coefficient between textural parameter evaluated by sensory evaluation and Instron (compression force) for cheese.

**Figure 7:**
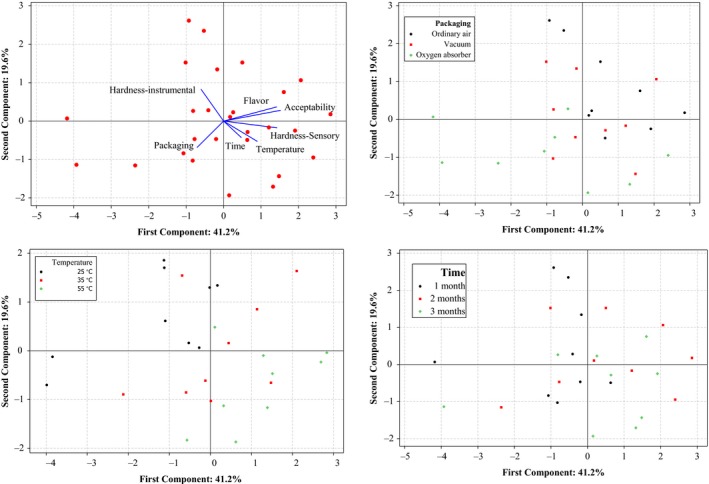
Principal component analysis biplot on properties of pistachio samples

Data on flavor, acceptability, sensory, and instrumental hardness of pistachio samples were subjected to PCA (Figure 7). The multivariate treatment of the data obtained for the samples permitted the reduction of the variables to two principal components, which together explained 60.7% of the total variability. The first axis accounted for 41.2% and the second axis for 19.6%. According to the PCA biplot, time, temperature, flavor, acceptability, and sensory hardness were positively correlated to PC1 axis, whereas packaging type and instrumental hardness were negatively correlated to PC1 axis. Flavor, acceptability, and instrumental hardness were positively correlated to PC2 axis, while sensory hardness, time, temperature, and packaging type were negatively correlated to PC2 axis. As clearly revealed in the PCA plot (Figure 7), acceptability and flavor of samples packed in ordinary air have been higher than other samples. Sensory hardness in samples packed in ordinary air and vacuum was higher than samples with oxygen absorber. Also, instrumental hardness in ordinary air samples was higher than other samples. Flavor, acceptability, and sensory hardness of samples were not significantly different after 2 and 3 months. This behavior was more evident in samples packed in ordinary air and stored at temperatures of 35 and 55°C. The highest scores of acceptability and flavor were observed in samples stored at 35°C. On the other hand, the highest sensory score of hardness and subsequently the softest texture was detected in samples stored at 55°C. Besides, considering the instrumental hardness of samples, it was found that the samples held at 25°C had the hardest texture. The effectiveness of PCA in analyzing correlation between textural parameters and sensory aspects in food technology from raw to final products such as roasted almonds (Varela, Chen, Fiszman, & Povey, 2006), dough and Barbari bread (Pourfarzad, Mohebbi, & Mazaheri‐Tehrani, 2012), dark chocolate (Owusu, Petersen, & Heimdal, 2013), sourdough bread (Najafi, Pourfarzad, Zahedi, Ahmadian‐Kouchaksaraie, & Khodaparast, 2016), and potato chips (Salvador, Varela, Sanz, & Fiszman, 2009) has been documented by different researchers. The instrumental test used in this study was able to estimate, to discriminate, and to predict quite sensibly sensory aspects. Additionally, texture analyzer is an instrument that extensively utilized for quality control of a wide variety of food substances, and food companies are familiar with it. This benefit makes it appropriate for industrial application.

## 4 CONCLUSION

The results of this study showed that the effect of both temperature and storage time was significant on hardness of raw dried pistachio nuts but at the term of sensory attributes was not significant. Moreover, pistachio hardness decreases with increasing storage time in atmosphere and oxygen scavenger packaging system. The results also indicated that the correlation between instrumental hardness and sensory properties (texture) was highly significant (*R* ^2^ = 0.9). PCA allowed discriminating among texture and sensory specialties. As well, it was proved that PCA is able to extract relevant information and offer an easy and talented method for the explanation of properties of pistachio samples.

## CONFLICT OF INTEREST

The authors declare that they do not have any conflict of interest.

## ETHICAL APPROVAL

Ethical Review: This study does not involve any human or animal testing.

## INFORMED CONSENT

Written informed consent was obtained from all study participants.
